# Development of a k-Nearest Neighbors Model for the Prediction of Late-Onset Alzheimer’s Risk by Combining Polygenic Risk Scores and Phenotypic Variables

**DOI:** 10.3390/genes16040377

**Published:** 2025-03-26

**Authors:** Sandra Ferreiro López, Rosana Ferrero, Jorge Blom-Dahl, Marta Alonso-Bernáldez, Adán González, Guillermo Pérez-Solero, Jair Tenorio-Castano

**Affiliations:** 1 ADNTRO Genetics, Carretera Betlem, s/n, Colonia de Sant Pere, 07579 Arta, Spain; darwin@adntro.com (S.F.L.); fireblom@adntro.com (J.B.-D.); nettie.stevens@adntro.com (M.A.-B.); watson@adntro.com (A.G.); morgan.monzon@adntro.com (G.P.-S.); 2 Center of Applied Ecology and Sustainability (CAPES), Pontificia Universidad Católica de Chile, Santiago 8331150, Chile; rosana.ferrero@gmail.com; 3 INGEMM, Institute of Medical and Molecular Genetics, La Paz University Hospital, IdiPAZ, 28046 Madrid, Spain; 4 ITHACA, European Research Network, La Paz University Hospital, 28046 Madrid, Spain; 5 Network for Biomedical Research on Rare Diseases (CIBERER), Carlos III Health Institute (ISCIII), 28046 Madrid, Spain

**Keywords:** Alzheimer’s disease, epidemic, genomic medicine, KNN, late-onset Alzheimer’s disease, machine learning model, polygenic risk score

## Abstract

Introduction: Alzheimer’s disease (AD), and more specifically late-onset Alzheimer’s disease (LOAD), represents a considerable challenge in terms of early and timely diagnosis and treatment. Early diagnosis is crucial to improve the efficacy of the therapies and patients’ quality of life. The current challenge is to accurately identify at-risk individuals before the manifestations of the first symptoms of AD. Methods and results: Here, we present an improved model for LOAD risk prediction, which applies the k-nearest neighbors (KNN) algorithm. We have achieved a sensitivity of 0.80 and an area under the curve (AUC) of 0.71, which represents a high performance especially when compared to an AUC of 0.66 reported previously in 2019 using a KNN model. Discussion: The application of a mathematical model that combines genetic and clinical covariates showed a good prediction of the AD/LOAD risk, with the higher weight being the polygenic genetic risk, *APOE* haplotype, and age. Compared to previous studies, our model integrates and correlates genetic prediction together with phenotypic information by fine-tuning the parameters of the model in order to achieve the best performance. This algorithm can be used in the general population and does not require the manifestation of any symptoms for its effective application. Thus, we present here an advanced model for risk prediction of LOAD.

## 1. Background

Alzheimer’s disease (AD) is nowadays considered an epidemic, which accounts for 60–80% of dementia individuals, a rate that is exponentially increasing, especially in elderly populations [1]. The prevalence of AD in Europe is estimated at 5.05%, being higher in females compared to males (7.13% vs. 3.31%) [2,3,4,5], which in general, increments with age, especially in individuals above 65 years, being 34% in individuals above 82 years old [3,4,6]. AD is more frequent in females compared with males, which is probably associated with life expectancy differences [3,4,5]. Although AD has a heterogeneous clinical presentation, the first symptom reported in individuals with AD is the accumulation of the β amyloid peptide, which finally leads to the appearance of senile plaques [4,7,8]. One of the consequences is the phosphorylation of the tau protein, which can correlate with the development of neurofibrillary tangles [6,7,9].

Therefore, the physiopathological mechanisms and features of AD include senile plaques, neurofibrillary tangles, vascular granule degeneration, neuronal loss, astrocyte gliosis, and amyloid angiopathy, among others [3,4,6]. At the clinical level, patients with AD will manifest cognitive impairment, usually starting with memory problems, aphasia, apraxia, agnosia, and alterations in normal activity behavior. In addition, progressive memory deterioration is one of the main symptoms detected in patients with AD [10] as well as feeding problems, which have been reported in up to 85% of the patients [11]. Several studies suggested that many years before the manifestation of the first symptoms of AD, the brain suffers biological changes in an apparently healthy individual, which is known as the preclinical AD stage [12,13]. AD clinical presentation can be subdivided into three stages according to severity: mild, moderate, and severe.

Currently, the treatments of AD are focused on delaying the progression of the neurocognitive features of the disease and addressing the cognitive and behavioral symptoms. These treatments included cholinesterase inhibitors and antagonists of the *N*-methyl-D-aspartate (memantine) [14,15,16,17]. Other drugs include aducanumab and lecanemab, whose function is to remove plaques and a form of β-amyloid called protofibrils that plays a role in the development of β-amyloid plaques [18].

Late-onset Alzheimer’s disease (LOAD) represents more than 90% of AD cases [18] and is associated with synaptic loss, which correlates with progressive cognitive impairment and neurodegeneration. Therefore, early diagnosis, especially for LOAD, is a crucial challenge for improving treatment outcomes [3,12] and anticipating clinical interventions. Despite the variety of current clinical diagnostic methods (MRI, PET, blood markers, phosphorylated tau protein, levels of β amyloid protein), none can accurately detect AD in early-stage patients without pathological changes [3,15,19]. At a genetic level, apolipoprotein E encoded by the *APOE* gene has been seen to be associated with a higher risk of development of AD. Specifically, allele ε4 of *APOE* is associated with a higher risk of AD development compared to alleles ε2 and ε3, giving an increased risk of between 3 to 12 times, and is also associated with a lower age onset of the disease [18,20,21]. Some authors have suggested that the association between allele ε4 and an increased risk of AD is due to the inhibition of amyloid-β (A-β) clearance, thereby promoting A-β aggregation [18]. Some other authors suggest that APOE ε4 influences tau pathology-mediated neurodegeneration and that it can also impact microglial responsiveness, lipid transportation, synaptic intercorrelation and plasticity, and glucose metabolism.

In this context, the increased development and application of machine learning (ML) techniques and models, such as k-nearest neighbors (KNN), can be used to combine a set of covariates in a way that allows the identification of individuals at high risk of developing LOAD before symptoms appear or before the identification of other metabolites, allowing for earlier intervention and better treatment outcomes. These models can combine both polygenic risk scores (PRS) with well-known clinical risk features [22]. ML is a subfield of artificial intelligence that allows the development of algorithms and models capable of learning patterns and creating predictions from the data of input. In AD, ML has been used to analyze large and complex datasets, such as genetic and neuroimaging data, in order to develop predictive models for the diagnosis, progression, and treatment of LOAD [23,24,25].

However, the challenge for ML models based on neuroimaging data is the need to analyze images of a diseased brain in already diagnosed patients who present symptoms. That is why genomics can support in early stages the identification of an individual’s risk of AD for earlier detection. Genetic analysis can help to identify individuals at risk even before they manifest the first symptoms, being able to maximize medical follow-up in those people at high risk for the early detection of the disease and a better stratification of the population.

There are two very different types of AD, early-onset (EOAD) and late-onset (LOAD) [26,27]. EOAD occurs in only 5% of AD cases and occurs in people younger than 65 years [26]. This type of AD is characterized by the presence of pathogenic variants in the *PSEN1*, *PSEN2*, and *APP* genes [28]. In contrast, LOAD is the most common type of AD that occurs in people over 65 years of age and is influenced by the *APOE* haplotype and multiple polymorphisms with a relatively low effect size, but the analysis of all of these indicators collectively confers a significant risk [20].

The estimated heritability of LOAD is around 60–80% according to previous studies [18,29], suggesting a significant genetic influence in susceptibility. This highlights the critical need to incorporate genetic polygenic scores that include polymorphism with relatively low effect sizes with *APOE* haplotype identification in the predictive models of LOAD [20]. Thus, in order to enhance the effectiveness of LOAD’s genetic predictive models, it is essential to incorporate phenotypic variables in the model. Clinical risk factors include age, sex, cholesterol, smoking, and pre-existing health conditions such as diabetes. Integrating these phenotypic variables with genetic markers is predicted to allow a more accurate approach to LOAD prediction.

The objective of this study is to develop an ML pilot model, based on KNN, and a methodology that can be useful to predict the risk of developing LOAD by combining genetic and phenotypic covariates. Predictive and statistical evaluation of these factors in individuals diagnosed with AD will complement current understanding and support the development of more sensitive and effective models.

## 2. Methods

### 2.1. Sample Selection

For the construction of the predictive model, both case and control samples from the UK Biobank (UKB) were obtained [30]. The UKB database is the product of a large-scale research project whose purpose is to promote advances in the prevention, diagnosis, and treatment of various serious diseases. Its relevance lies in the merging of clinical and genomic data from more than 500,000 participants over 40 years old at the age of recruitment. Their extensive phenotypic and genotypic data were collected at the time of recruitment at 22 assessment centers in England, Scotland, and Wales between 2006 and 2010.

Descriptive statistics of the groups selected for this study can be found in Supplementary Materials Tables S1–S7. For the cases, 2840 samples were selected based on the criteria existence of a date in the field “Date of Alzheimer’s disease report” and “age at recruitment” between 60 and 99 years, thus limiting our study to LOAD. For the controls, individuals with Alzheimer’s were excluded and the same age range for cases was applied for controls. In addition, we decided to exclude from the controls individuals diagnosed with any type of dementia different from Alzheimer’s. A total of 210,626 individuals met the criteria applied for the control cohort. Given the large difference in the “*n*” between the two groups, we decided to randomly select only close to 10,000 individuals to develop and test our pilot algorithm.

### 2.2. Variables Studied

In the realm of genetic epidemiology, the identification and quantification of genetic variants associated with AD are paramount. For SNP selection, we have used the findings of De la Fuente, Javier, et al. (2022) [23]. These findings are based on individuals diagnosed with AD and those with a family history of AD across several cohorts, including UKB, IGAP, PGC-ALZ, and ADSP. Further details can be found in the original articles [31,32].

Among the polymorphisms that have demonstrated a statistically significant correlation with the etiology of AD, we have included in our analysis only those that were present on the genotype repository from the UKB genotyping data eschewing imputed variants to maintain data integrity. These SNPs were subjected to quality control (detailed in the “Quality Filtering” section).

In operationalizing the influence of SNPs, we synthesized a composite score for each individual was computed as follows:

score = (Σ(g_i + 1) × β_i − μ_control)/σ_control

where:

g_i represents the genotype encoding for the ith SNP (1 for protective homozygous, 2 for heterozygous, and 3 for risk homozygous);

β_i is the β coefficient for the ith SNP from GWAS summary statistics;

μ_control is the mean z-score of the control group;

σ_control is the standard deviation of the control group.

This formulation ensures non-zero entries in our input matrix and reflects the relative effect sizes derived from GWAS.

Further to these genetic markers, we included the apolipoprotein E (APOE) haplotype, encompassing rs429358 and rs7412 polymorphisms, as a critical genetic predictor for AD, attributable to more than fifty percent of AD manifestation [33]. To integrate the APOE haplotypes within our multivariate prognostic model, we employed an ordinal encoding strategy based on the conferred risk magnitude:

Lower risk: Haplotypes ε2/ε2 and ε2/ε3 were encoded as −1.

Standard risk: The ε3/ε3 haplotype was assigned a value of 0.

Elevated risk: Haplotypes ε2/ε4 and ε3/ε4 were encoded as 1.

High risk: The ε4/ε4 haplotype was assigned a value of 2.

To bolster the predictive capability of our model, we integrated phenotypic risk factors gleaned from the extant literature. The encoding schema for these variables is bifurcated into qualitative and quantitative measures, as follows:

Qualitative variables (smoking status, diabetes mellitus, and gender) were encoded binarily (0 for absence of risk factor; 1 for presence of risk factor, and in the case of gender, 0 for women and 1 for men).

Quantitative variables (age and cholesterol levels) were incorporated directly with their numerical values.

Normalization of the data was executed using the scale () function in R, to ensure uniformity of variable scales. A comprehensive elucidation of the model’s predictive variables is detailed in the Supplementary Materials Tables S1–S7.

### 2.3. Quality Filtering—Final Dataset Selection

Genotypic information underwent thorough rigorous quality control measures (QC—used in previous similar articles [8]), which led to the exclusion of data based on the following criteria:

SNPs with zero variance: Single nucleotide polymorphisms without variance mean that the allele does not vary across the studied population. Including SNPs without variance in genetic association studies is not informative because they do not contribute to genetic diversity or disease association.

SNPs with a missing genotype rate greater than 10%: A high missing genotype rate indicates that for a significant portion of the population, the genetic information at that SNP is unknown. This ensures that the analysis is based on SNPs with robust and complete genetic data across the studied samples.

SNPs with a minor allele frequency (MAF) lower than 0.01: Minor allele frequency refers to the frequency at which the less common allele occurs in the population. The exclusion of SNPs with very low MAFs is often carried out to focus the study on genetic variations that are more common in the population, as rare variants may not have enough statistical power to detect an association with diseases or traits.

Individuals with more than 10% of genotypes absent: Excluding individuals with a significant proportion of missing genotypes ensures the integrity of the data set. This minimizes potential errors and biases in the analysis that could arise from incomplete genetic information.

After implementing these quality controls, the total number of SNPs was reduced to 379. Likewise, the number of cases and controls decreased to 2547 and 8699 individuals, respectively.

No LD analysis was implemented in common with the original articles for the corresponding summary statistics [31,32].

### 2.4. Pipeline for Evaluation of the Model

**Figure 1:**
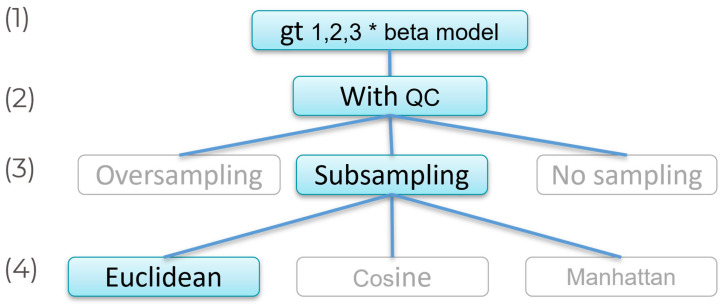
Diagram of the different technical combinations evaluated in the predictive model. The decision algorithms that maximize the metrics of the model and those selected for model refinement are highlighted in blue. (1) Multiply the number of risk alleles by the effect size of the SNP. Likewise, we coded protective homozygotes as 1, heterozygotes as 2, and risk homozygotes as 3 to avoid the existence of 0 in the input matrix. (2) We then applied quality filtering on our SNPs and individuals to work only with quality data despite this step slightly reducing the number of SNPs (*n* = 379). (3) As shown in Table 1, results favor the subsampling technique for balancing the training dataset. (4) Evaluation of different distances to calculate genetic distance.

To construct our predictive model, we have employed the KNN algorithm due to its versatility in handling both classification and regression tasks. The KNN algorithm, a non-parametric method, is particularly advantageous for datasets with complex or undefined distributions, as it does not rely on underlying data assumptions (Figure 1).

The effectiveness of KNN hinges on the selection of k, the number of nearest neighbors considered for making predictions. This hyperparameter was optimized using the R package “caret”, employing a 5-fold cross-validation technique to evaluate 60 distinct k values, thereby enhancing the model’s predictive accuracy. Furthermore, the model’s binary classification threshold, critical for distinguishing between the two classes, was finely tuned to balance sensitivity and specificity. This optimization was conducted using the version 1.1.5 “KernelKnn” package in R, which facilitates threshold adjustments to achieve an optimal trade-off between true positive rate (sensitivity) and true negative rate (specificity) by taking into consideration the probability value provided by the model.

The dataset was then partitioned into training (70%) and testing (30%) subsets to train and validate the KNN model’s performance. Given the potential class imbalance in the training set, we applied oversampling and subsampling techniques to ensure a balanced representation of classes, thereby improving model reliability.

Our evaluation encompassed seven distinct predictive models, initially focusing on single nucleotide polymorphisms (SNPs) to assess various sampling strategies (models 1–3) and distance metrics (models 3–5) for their impact on model performance. The optimal model was selected based on a comprehensive analysis of sensitivity, specificity, and the receiver operating characteristic (ROC) curve, aiming to maximize predictive effectiveness.

### 2.5. Sampling Techniques

To mitigate the skewed distribution of AD cases within the training dataset, which consisted of 23% cases and 77% controls, we employed three distinct sampling strategies to balance the data representation (referenced as models 1–3 in Table 1) (Figure 1):

No Sampling Approach: In this method, no sampling techniques were employed, thereby maintaining the original distribution of the dataset. Consequently, the training set encompassed 7851 participants, with the test set comprising 3395 participants, maintaining the case–control ratio at 23:77.

Oversampling Technique: To achieve a balanced distribution, this method involved augmenting the minority class (cases) through random duplication until parity with the control group was reached. This adjustment resulted in a balanced training set of 12,148 participants, with an equal 50:50 distribution between cases and controls.

Subsampling Method: This approach entailed reducing the number of participants in the majority class (controls) within the training set to align with the case count, leading to a balanced cohort of 3556 participants, with a 50:50 ratio between cases and controls.

**Table 1:** Information on the different models evaluated throughout the project. This table describes the sampling techniques used, the distances, the observations of the model, the cut-off points for classifying a new individual as a case or control, the number of samples used to train and test the model, the number of cases and controls, and specifically, the metrics for sensitivity, specificity, and AUC. The sample technique to balance the training dataset with the best results was the subsampling technique and the distance that best fits our dataset was Euclidean. When we consider all parameters, model 7 is the one that maximizes the sensitivity of the model while maintaining a reasonable specificity.

Information on the Different Models Evaluated											
Model	Sampling	Distance	Observations	Threshold	Test	Train	Cases	Controls	SEN *	SPEC *	AUC
Model 1	No sampling	Euclidean	379 SNPs	0.50	3395	7851	2547	8699	0.12	0.99	0.56
Model 2	Over sampling	Euclidean	379 SNPs	0.50	3395	12,148	6075	8699	0.56	0.62	0.59
Model 3	Subsampling	Euclidean	379 SNPs	0.50	3395	3556	2547	4404	0.64	0.72	0.68
Model 4	Subsampling	Manhattan	379 SNPs	0.50	3395	3556	2547	4404	0.60	0.72	0.68
Model 5	Subsampling	Cosine	379 SNPs	0.50	3395	3556	2547	4404	0.48	0.52	0.50
Model 6	Subsampling	Euclidean	Z-score, APOE, and phenotypic data **	0.50	3235	3348	2394	4196	0.69	0.74	0.71
Model 7	Subsampling	Euclidean	Z-score, APOE, and phenotypic data **	0.42	3235	3348	2394	4196	0.80	0.61	0.71

* SEN = sensitivity; SPEC = specificity, ** smoker, diabetes, cholesterol, age and sex.

Furthermore, in models incorporating both genetic and phenotypic variables, the occurrence of missing data (NA) within the phenotypic variables required a slight reduction in the sample size. This adjustment was due to the absence of comprehensive variable information across all evaluated samples, resulting in a refined dataset encompassing 2394 cases and 4189 controls for the development of our final algorithm (as detailed in models 6–7 of Table 1).

This refined approach underscores our commitment to methodological rigor and the pursuit of scientific accuracy, ensuring that our model’s development is grounded in robust and balanced data analysis techniques.

### 2.6. Distances

We also explored various metrics to quantify the dissimilarity between the genetic profiles of the samples under prediction and those within our trained dataset. Specifically, we assessed three distinct distance measures to evaluate their efficacy in the genotype-based model (referenced in models 3–5 of Table 1):

Euclidean Distance: This metric, the most prevalent in quantitative genetic analysis, serves as the default option in numerous R statistical packages. It was used as the benchmark distance measure for appraising the effectiveness of different sampling strategies outlined in models 1–3. The Euclidean distance calculates the root of square differences between the coordinates of a pair of objects.

Cosine Similarity: Given the specific context of the data analyzed, we also considered the cosine similarity measure, which interprets angular differences as a measure of dissimilarity between vectors. This metric is particularly useful in high-dimensional spaces, offering insights into the orientation rather than the magnitude of data points.

Manhattan Distance: Acknowledging the binary nature of our classification task—distinguishing between healthy and diseased states—we explored the potential of the Manhattan distance to enhance the model’s sensitivity. This metric, summing the absolute differences between points in a space, is often favored for categorical or binary data due to its alignment with the lattice structure of such datasets.

These distance metrics were rigorously evaluated for their impact on the model’s performance, with the objective of optimizing the algorithm’s sensitivity and overall predictive accuracy in the context of binary classification tasks.

### 2.7. Statistical Evaluation of the Multivariate Predictive Model

We evaluated several performance metrics described by the confusion matrix such as accuracy, sensitivity, specificity, ROC curve, precision, and F1 score. Finally, we compared the performance of each model based on the sensitivity, specificity, and ROC curve. We had a special interest in the sensitivity metric as our goal was to maximize the number of correctly identified positive cases with AD within our model. We use the specificity metric in order to avoid losing the balance of the model performance but at the same time, correctly detect cases and controls.

Finally, we also evaluated the area under the ROC curve since it is a metric that assesses the overall performance of the model, considering all possible combinations of sensitivity and specificity. The higher the value of the AUC-ROC, the better the performance of the model.

### 2.8. Statistical Analysis of the Variables

In order to determine whether there are statistically significant differences between case–control groups in relation to each predictor variable, we applied statistical hypothesis testing. For numerical predictors (Z-score, age, and cholesterol), we evaluated the statistical assumptions necessary to select the type of test (normal and symmetrical distribution) and we assumed unequal variances (heterogeneity of variance). For categorical predictors (*APOE*, sex, diabetes, and smoking), we applied the Chi-square independence statistical test.

## 3. Results

### 3.1. Technical Evaluation of the Model

A cohort of 11,246 UKB participants, aged between 60 and 99 years, was evaluated by different predictive models with the aim of optimizing the sensitivity of the multivariate algorithm.

Table 1 shows the predictive models adjusted for SNP predictors (models 1–5) and the combination of genetic and phenotypic predictors (models 6–7) according to the sampling techniques used to balance the training dataset (over- and subsampling), and the different distances used (Euclidean, cosine, and Manhattan). In terms of sensitivity, models that combine genetic and phenotypic variables (Table 1, models 6 and 7) outperformed models composed only of polymorphisms associated with LOAD (models 1–5).

Both oversampling and subsampling improved the metrics obtained in the untreated data, demonstrating an improvement in the algorithm when balancing the groups in the training dataset (50% cases and 50% controls). The maximum resolution was obtained with the subsampling technique (Table 1, model 3) where the cases of the initial dataset (2547) were maintained, and the number of controls was reduced in order to balance the training dataset (4404). With this strategy, a sensitivity of 64%, a specificity of 72%, and an ROC curve of 68% were obtained.

Then, using the subsampling technique, different distances used by the algorithm were evaluated. Euclidean distance (Table 1, model 3) performed best (sensitivity = 64%, specificity = 72%, AUC = 68%), followed by Manhattan distance (Table 1, model 4) (sensitivity = 60%, specificity = 74%, AUC = 67%). Cosine distance (Table 1, model 5) was undoubtedly the approach that provided the worst results (sensitivity = 48%, specificity = 52%, AUC = 50%).

The best model was the one that included both genetic and phenotypic variables, increasing sensitivity to 69%, with a specificity of 74% and an AUC of 71% (Table 1, model 6).

**Figure 2:**
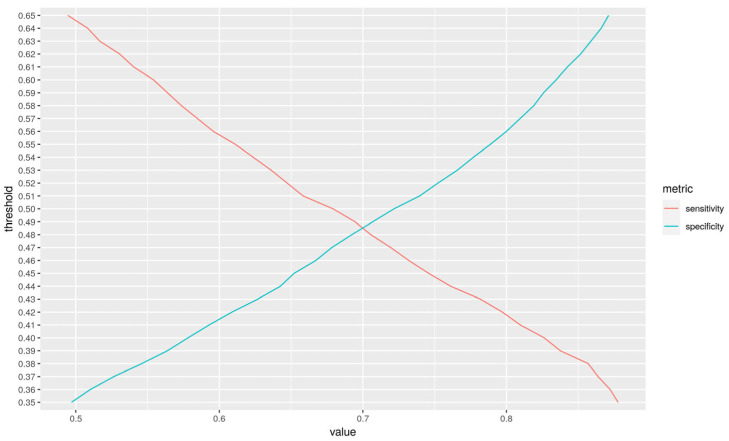
Evaluation of the sensitivity and specificity of our model by varying the cut-off threshold by the algorithm to classify an individual as healthy or with disease. The sensitivity of the model is highlighted in red and the specificity in blue. By modifying the cut-off point of our algorithm, we increased the ability of our model to detect people with Alzheimer’s disease (sensitivity = 80%, specificity = 61%, AUC = 71%) (Table 1-model 7).

For the latter model, analysis of the optimal cutoff point for classifying individuals as cases or controls maximized sensitivity (Table 1, model 7) by applying a probability of 0.42 (Figure 2) while maintaining a specificity of 61%.

### 3.2. Statistical Evaluation of Model Variables

Once our algorithm was developed, we decided to explore the impact of these factors on the incidence of AD using statistical inference.

**Figure 3:**
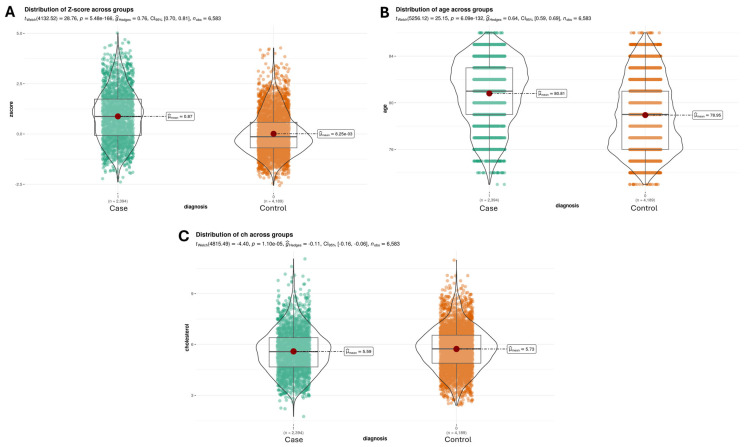
Statistical evaluation of the numerical variables for the different classes. (**A**) There is a statistically significant difference (*p* = 5.48 × 10^−166^) between cases and controls for the “Z-score” variable. The gHedges value indicates that the effect size of this covariate is large (0.76); (**B**) There is a statistically significant difference (*p* = 6.098 × 10^−132^) between cases and controls for the variable “age”. The gHedges value indicates that the effect size of this covariate is medium (0.64); (**C**) There is a statistically significant difference (*p* = 1.10 × 10^−5^) between cases and controls for the predictor “cholesterol”. The gHedges value indicates that the effect size of this variable is small and negative (−0.11).

When evaluating the effect of the Z-score on the presence of AD (Figure 3A), we observed large effect sizes (gHedges = 0.76) and statistically significant differences compared to the control group (*p* = 5.48 × 10^−166^). Regarding the differences in the age (Figure 3B) of the subjects between the group with and without Alzheimer’s, we found statistically significant differences (*p* = 6.098 × 10^−132^) and large effect sizes (gHedges = 0.64). However, the differences in cholesterol values (Figure 3C) between control subjects and subjects with Alzheimer’s were small (gHedges = −0.11) but again statistically significant (*p* = 1.10 × 10^−5^).

**Figure 4:**
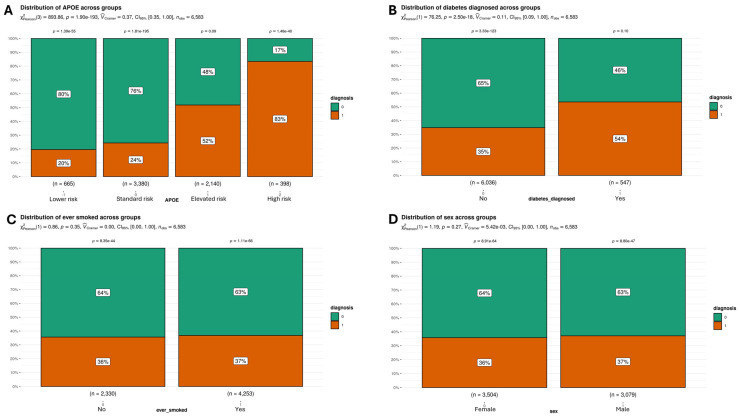
Statistical evaluation of categorical predictors for different classes. (**A**) When the different groups of APOE are evaluated together, statistically significant differences (*p* = 1.90 × 10^−193^) were observed between cases and controls. The value of Vcramer indicates that the strength of association of this predictor is medium (0.37). If we focus on each group individually, there is a statistically significant difference for all groups except for group 1—“Elevated risk” (ε2/ε4 and ε3/ε4). The percentage of subjects with Alzheimer’s was higher only in group 2—“High risk” (ε4/ε4); (**B**) We also found statistically significant differences (*p* = 2.50 × 10^−18^) between cases and controls for the predictor “diabetes”. The value of Vcramer indicates that the strength of association of this predictor is small (0.11); (**C**) We did not observe statistically significant differences (*p* = 0.35) between cases and controls for the predictor “smoker”; (**D**) We did not find statistically significant differences (*p* = 0.27) between cases and controls for the variable “sex”.

Regarding categorical variables, we detected small (Vcramer = 0.11) statistically significant differences (*p* = 2.50 × 10^−18^) for diabetes (Figure 4B), where subjects with diagnosed diabetes had a higher percentage of incidences of Alzheimer’s (54% compared to 35% of subjects without diabetes with Alzheimer’s). We did not find statistically significant differences for either smoking (Figure 4C) (*p* = 0.35) or sex (Figure 4D) (*p* = 0.27). Regarding the evaluation of the different *APOE* haplotypes collectively (Figure 4A), we detected statistically significant differences (*p* = 1.90 × 10^−193^) with a medium strength (Vcramer = 0.37). When analyzing Figure 4A in detail, we see that only the group categorized as 1—“Elevated risk” (ε2/ε4 and ε3/ε4)—did not present differences between control/case classes (*p* = 0.09). The percentage of subjects without Alzheimer’s was significantly higher in groups −1—“Lower risk” (ε2/ε2 and ε2/ε3)—(80%, *p* = 1.39 × 10^−55^) and 0—“Standard risk” (ε3/ε3)—(76%, *p* = 1.81 × 10^−195^) while for haplotype 2—“High risk” (ε4/ε4)—a significant but inverse pattern was observed (only 17% of healthy subjects, *p* = 1.48 × 10^−40^).

According to the data derived from the sampling, the covariates that showed the most pronounced differences between the groups with and without Alzheimer’s were Z-score (*p* = 5.48 × 10^−166^), *APOE* (*p* = 1.90 × 10^−193^), and age (*p* = 6.09 × 10^−132^). In contrast, variables related to gender (*p* = 0.27) and smoking (*p* = 0.35) did not show significant differences (Figure 3 and Figure 4).

## 4. Discussion

Alzheimer’s Disease (AD) is the most prevalent dementia in the population and the incidence has been growing, especially in recent years. The US Alzheimer’s Association estimated a proportion of 6.9 million older adults with AD in 2024, which represents an increase of 0.83 million people compared to 2020 and it is estimated that by 2060, this number will increase to 13.85 million individuals. Due to the health impact of the disease and the bad prognosis of individuals with AD, developing new strategies for early detection and prevention are required.

In our investigation, we have constructed a machine learning (ML)-enhanced predictive framework for the precocious identification of late-onset Alzheimer’s disease (LOAD), a pivotal step in augmenting the therapeutic outcomes of interventions. The empirical evaluation of our model revealed commendable performance metrics: sensitivity (true positive rate) at 0.80, specificity (true negative rate) at 0.61, and an area under the receiver operating characteristic curve (AUC-ROC) of 0.71. Such efficacious outcomes stem from a meticulous methodology based on quality control, optimized subsampling, Euclidean distance calculations, and strategic selection of predictive variables. A critical aspect of our model’s success is the Euclidean distance, the subsampling technique, and the integration of genetic and phenotypic data, which, as evidenced in Table 1, significantly bolsters predictive accuracy.

Among the phenotypic covariates, variables such as Z-score, *APOE* genotype, and age exhibited pronounced differential characteristics between the Alzheimer’s-affected and non-affected groups. Notably, cholesterol levels, expressed in millimoles per liter (mmol/L), presented marginally lower averages in the Alzheimer’s group compared to the control group. This observation could be attributed to potential interactions with other variables or the possibility that cholesterol levels may not constitute a significant risk factor for AD, a hypothesis that remains contentious in the existing literature and requires further studies to confirm this hypothesis.

The incorporation of polygenic risk scores (PRS) into clinical practice offers several advantages for healthcare systems and providers. First, PRS-based tools enable the stratification of individuals based on their genetic predisposition to LOAD, facilitating early intervention strategies that can significantly alter disease progression and patient outcomes. Unlike conventional diagnostic modalities, such as neuroimaging, which require pathological changes to have manifested, PRS can identify at-risk individuals well before clinical symptoms emerge, offering a window for preemptive therapeutic measures and preventive interventions.

Furthermore, PRS methodologies underscore a personalized medicine approach, tailoring prevention and treatment strategies to the individual’s genetic makeup. This precision in healthcare delivery not only enhances patient care but also optimizes resource allocation within healthcare systems, reducing the economic burden associated with late-stage disease management.

### 4.1. An ML Classifier Based on Genetic and Phenotypic Characteristics: Performance Comparison with Previous Models

Our model shows a significant improvement compared to the KNN algorithm benchmark reported in a 2021 study (63.8% sensitivity) [24,25]. These improvements are attributed to strategic technical decisions and the inclusion of additional phenotypic variants in our model, reflecting a significant advance in the accuracy and applicability of the model. Although the performance of our model may be lower than those that include images, our approach is different and differential, focusing not on a definitive diagnosis, but on the evaluation of existing risk based solely on genetics and easily assessed phenotypic risk variables [34].

The importance of the different approaches adopted to optimize our model is considerable (sampling techniques, distance of the algorithm, QC, and input provided). In particular, we have accounted for the effect of different single nucleotide polymorphisms by multiplying the number of risk alleles by their associated weight. This method has allowed a better interpretation and application of genetic data, contributing significantly to the accuracy of the model.

Regarding the variables incorporated in the model, they are present both in people with and without symptoms of Alzheimer’s disease. It is not necessary to wait until subjects develop characteristic symptoms of the pathology, allowing a more detailed medical follow-up and early diagnosis of the disease. Additionally, by combining genetic and phenotypic variables, we increase the scope of our model.

Further analysis is required to analyze the sensitivity and specificity of this model in other AD forms, such as early onset AD, hippocampal-sparing AD, or limbic-predominant AD. Larger cohorts with different etiology of AD can be studied as an exploratory to study the performance of the model in those populations.

### 4.2. Applicability and Clinical Utility

This algorithm assesses the genetic risk of developing Alzheimer’s with a sensitivity of 80%. In other words, it has the ability to correctly detect 80% of AD patients, making it possible to assess the genetic risk of a population for subsequent stratification.

By applying this predictive model, it would be possible to recognize those people with a higher risk of developing Alzheimer’s and intensify their follow-up to make an early diagnosis and apply the appropriate treatment to maximize its effectiveness.

### 4.3. Limitations and Future Lines of Research

This study highlights the importance of combining genetic and phenotypic variables to assess the risk of LOAD with high sensitivity (80%). More specifically, the study highlights the great importance of the variables Z-score, *APOE* genotype, and age. Currently, we are at the stage of exploring the use of more complex ML algorithms to explore the possibility of increasing the sensitivity of our model. The intention is to follow the same methodology but take advantage of the advanced capabilities of these algorithms to further improve the accuracy of our predictions.

A consideration for future research could be the extension of our model to other databases, such as the Alzheimer’s Disease Neuroimaging Initiative (ADNI-https://adni.loni.usc.edu/) used by the paper used as the benchmark. It could be beneficial to explore this step in subsequent studies in order to verify the robustness, applicability of the model in other cohorts, and the transversality of the model.

In summary, our study not only showcases the potential of ML and PRS in early LOAD detection but also highlights the transformative impact such technologies could have on clinical practices, emphasizing early diagnosis, personalized treatment plans, and ultimately, improved patient care outcomes in the context of Alzheimer’s disease.

## Data Availability

All data for this project were obtained from the UK Biobank through application number 93016. The original contributions presented in this study are included in the article/Supplementary Material. Further inquiries can be directed to the corresponding author.

## Supplementary Materials

The following supporting information can be downloaded at: https://www.mdpi.com/article/10.3390/genes16040377/s1, Figure S1: Project diagram; Table S1: Descriptive of the APOE categorical variable in cases and controls; Table S2: Descriptive of the numerical variable Z-score in cases and controls; Table S3: Descriptive of the numerical variable diabetes in cases and controls; Table S4: Descriptive of the numerical variable age in cases and controls; Table S5: Descriptive of the categorical variable sex in cases and controls; Table S6: Descriptive of the categorical variable smoker in cases and controls; Table S7: Descriptive of the cholesterol variable (mmol/L) in cases and controls.
