# Supplementary material for: Development of a k-Nearest Neighbors Model for the Prediction of Late-Onset Alzheimer’s Risk by Combining Polygenic Risk Scores and Phenotypic Variables
Source: Genes (Basel). 2025 Mar 26;16(4):377. doi: 10.3390/genes16040377 (PMC12027161; doi:10.3390/genes16040377)
Supplement: Supplementary file 1 [file genes-16-00377-s001.zip › genes-3498336-supplementary.pdf]

Supplementary Material: Development of a k-Nearest Neighbors Model for the Prediction of Late-Onset Alzheimer's Risk by Combining Polygenic Risk Scores and Phenotypic Variables

Supplementary Material and Methods

Quality filtering

Genotypic information underwent thorough rigorous quality control measures (QC - used in previous similar articles), which led to the exclusion of data based on the following criteria (Figure 1):

- **SNPs with zero variance**: Single nucleotide polymorphisms without variance mean that the allele does not vary across the studied population. Including SNPs without variance in genetic association studies is not informative because they do not contribute to genetic diversity or disease association.
- **SNPs with a missing genotype rate greater than 10%**: A high missing genotype rate indicates that for a significant portion of the population, the genetic information at that SNP is unknown. It ensures that the analysis is based on SNPs with robust and complete genetic data across the studied samples.
- **SNPs with a minor allele frequency (MAF) lower than 0.01**: Minor allele frequency refers to the frequency at which the less common allele occurs in the population. The exclusion of SNPs with very low MAF is often done to focus the study on genetic variations that are more common in the population, as rare variants may not have enough statistical power to detect an association with diseases or traits.
- **Individuals with more than 10% of genotypes absent**: Excluding individuals with a significant proportion of missing genotypes ensures the integrity of the data set. It minimizes potential errors and biases in the analysis that could arise from incomplete genetic information.

After implementing these quality controls, the total number of SNPs was reduced to 379. Likewise, the number of cases and controls decreased to 2,547 and 8,699 individuals, respectively.

Sampling techniques

To mitigate the skewed distribution of AD cases within the training dataset, which consisted of 23% cases and 77% controls, we employed three distinct sampling strategies to balance the data representation (referenced as models 1-3 in Table 1):

- **No Sampling Approach:** We opted not to implement any sampling techniques, thereby maintaining the original distribution of the dataset. Consequently, the training set encompassed

7,851 participants, with the test set comprising 3,395 participants, maintaining the case-control ratio at 23:77.

- **Oversampling Technique:** To achieve a balanced distribution, this method involved augmenting the minority class (cases) through random duplication until parity with the control group was reached. This adjustment resulted in a balanced training set of 12,148 participants, with an equal 50% distribution between cases and controls.
- **Subsampling Method:** This approach entailed reducing the number of participants in the majority class (controls) within the training set to align with the case count, leading to a balanced cohort of 3,556 participants, with a 50:50 ratio between cases and controls (Figure 1).

Furthermore, in models incorporating both genetic and phenotypic variables, the occurrence of missing data (NA) within the phenotypic variables required a slight reduction in the sample size. This adjustment was due to the absence of comprehensive variable information across all evaluated samples, resulting in a refined dataset encompassing 2,394 cases and 4,189 controls for the development of our final algorithm (as detailed in models 6-7 of Table 1).

This refined approach underscores our commitment to methodological rigor and the pursuit of scientific accuracy, ensuring that our model's development is grounded in robust and balanced data analysis techniques.

Supplementary tables and figures

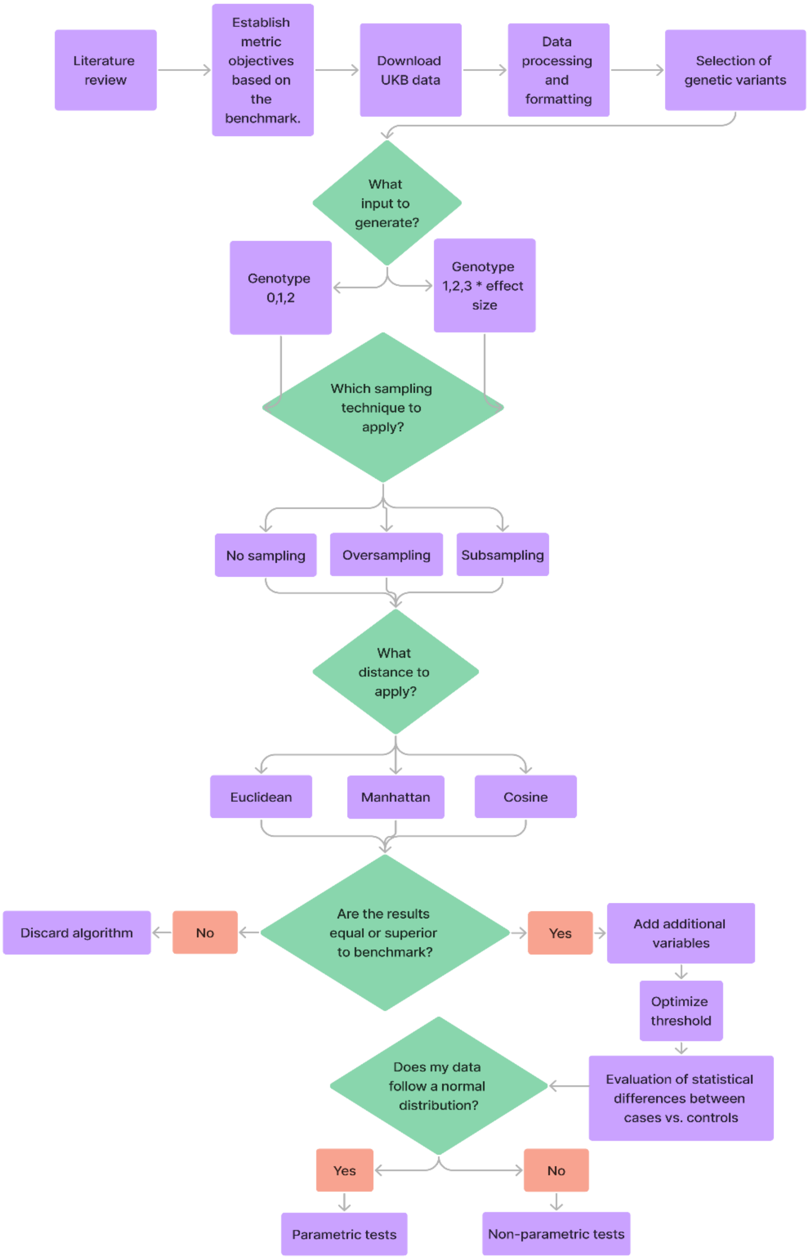

Supplementary Figure S1. Project diagram. From downloading the UKB data to obtaining results and evaluating the variables.

Group	Lower	Standard	Elevated	High
Cases	5%	34%	46%	14%
Control	13%	61%	24%	2%

Supplementary Table S1. Descriptive of the *APOE* categorical variable in cases and controls

Group	Mean	SD
Cases	0.874	1.262
Controls	0.0006	1.002

Supplementary Table S2. Descriptive of the numerical variable Z-score in cases and controls.

Group	Percentage diabetes	Count diabetes
Cases	12.23%	293
Controls	6.30%	519

Supplementary Table S3. Descriptive of the numerical variable diabetes in cases and controls.

Group	Mean	SD
Cases	80.81	2.81
Controls	78.94	2.99

Supplementary Table S4. Descriptive of the numerical variable age in cases and controls.

Group	Percentage female	Count Women
Cases	52.3%	1,253
Controls	53.59%	4,415

Supplementary Table S5. Descriptive of the categorical variable sex in cases and controls.

Group	Percentage smokers	Count smokers
Cases	65%	1,564
Controls	64%	5,342

Supplementary Table S6. Descriptive of the categorical variable smoker in cases and controls.

Group	Mean	SD
Cases	5.587	1.283
Controls	5.733	1.218

Supplementary Table S7. Descriptive of the cholesterol variable (mmol/L) in cases and controls.
